# The Interaction of *BDNF* and *NTRK2* Gene Increases the Susceptibility of Paranoid Schizophrenia

**DOI:** 10.1371/journal.pone.0074264

**Published:** 2013-09-17

**Authors:** Zheng Lin, Yousong Su, Chengfang Zhang, Mengjuan Xing, Wenhua Ding, Liwei Liao, Yangtai Guan, Zezhi Li, Donghong Cui

**Affiliations:** 1 Department of Psychiatry, Second Affiliated Hospital of Zhejiang University School of Medicine, Hangzhou, Zhejiang, China; 2 Shanghai Mental Health Center, Shanghai Jiao Tong University School of Medicine, Shanghai, China; 3 Department of Neurology, Shanghai Changhai Hospital, Secondary Military Medical University, Shanghai, China; 4 Bio-X Institute, Shanghai Jiao Tong University School of Medicine, Shanghai, China; Baylor College of Medicine, United States of America

## Abstract

The association between *BDNF* gene functional Val66Met polymorphism rs6265 and the schizophrenia is far from being consistent. In addition to the heterogeneous in schizophrenia per se leading to the inconsistent results, the interaction among multi-genes is probably playing the main role in the pathogenesis of schizophrenia, but not a single gene. Neurotrophic tyrosine kinase receptor 2 (NTRK2) is the high-affinity receptor of BDNF, and was reported to be associated with mood disorders, though no literature reported the association with schizophrenia. Thus, in the present study, total 402 patients with paranoid schizophrenia (the most common subtype of schizophrenia) and matched 406 healthy controls were recruited to investigate the role of rs6265 in *BDNF*, three polymorphisms in *NTRK2* gene (rs1387923, rs2769605 and rs1565445) and their interaction in the susceptibility to paranoid schizophrenia in a Chinese Han population. We did not observe significant differences in allele and genotype frequencies between patients and healthy controls for all four polymorphisms separately. The haplotype analysis also showed no association between haplotype of *NTRK2* genes (rs1387923, rs2769605, and rs1565445) and paranoid schizophrenia. However, we found the association between the interaction of *BDNF* and *NTRK2* with paranoid schizophrenia by using the MDR method followed by conventional statistical analysis. The best gene-gene interaction model was a three-locus model (*BDNF* rs6265, *NTRK2* rs1387923 and *NTRK2* rs2769605), in which one low-risk and three high-risk four-locus genotype combinations were identified. Our findings implied that single polymorphism of rs6265 rs1387923, rs2769605, and rs1565445 in *BDNF* and *NTRK2* were not associated with the development of paranoid schizophrenia in a Han population, however, the interaction of *BDNF* and *NTRK2* genes polymorphisms (*BDNF*-rs6265, *NTRK2*-rs1387923 and *NTRK2*-rs2769605) may be involved in the susceptibility to paranoid schizophrenia.

## Introduction

Schizophrenia is a chronic, recurrent, disabling mental disease with a high cost to society and individuals across the world [1]–[2]. Although the underlying etiology of schizophrenia is still poorly understood, lines of evidence suggests that dysfunction in neurodevelopmental processes resulting from genetic and environmental factors, contributes to the development of schizophrenia [3]–[4]. Brain-derived neurotrophic factor (BDNF), a member of the neurotrophin superfamily, plays important roles in various neurodevelopmental processes of the central nervous system (CNS), including neuronal differentiation and survival, synaptic connections as well as plasticity [5]–[6]. Mounting evidence has demonstrated that BDNF were involved in the pathophysiology of schizophrenia. Recently, Zhang et al. showed that BDNF levels were significantly lower in drug-free patients with schizophrenia [7]. Lee et al. also demonstrated that BDNF levels decreased significantly in unmedicated schizophrenic patients and elevated after successful antipsychotic treatment which parallel symptom improvement of the patients [8]. Furthermore, increasing postmortem studies have shown that BDNF levels were significantly lower in prefrontal cortex of schizophrenia patients [9]–[10]. In addition, it is notable that BDNF most likely functions through its high-affinity receptor, neurotrophic tyrosine kinase receptor 2 (NTRK2) [11]; and NTRK2 has also been found decreased in postmortem of schizophrenic subjects [12]. More interestingly, some previous study have also demonstrated that BDNF and NTRK levels were both decreased in the brain tissue of schizophrenic patients [13]–[14]. All these evidence suggest that dysfunction of BDNF and TrkB may be involved in the pathophysiology underlying schizophrenia.

At the molecular level, position 196 in exon 5 of the *BDNF* gene contains a G to A transition leading to an amino acid substitution (valine to methionine) at codon 66 in the precursor *BDNF* peptide sequence (dbSNP: rs6265). This polymorphism can affect activity-dependent secretion of BDNF, hippocampal function and morphology [15]–[16]. Thus, more and more research was carried out to explore the role of *BDNF* functional polymorphism rs6265 in development of schizophrenia. Muglia et al. found the association between this functional SNP and schizophrenia in Italian subjects [17]. Neves-Pereira et al. also showed a positive association in Caucasian [18]. The same result was also found in Chinese populations [19]. However, some literatures reported the contrary results, and the association studies between the functional SNP Val66Met and schizophrenia is far from being consistent [20]–[22].

As to *NTRK2* gene polymorphisms, previous studies have shown that *NTRK2* gene polymorphisms rs2769605, rs1387923, and rs1565445 were associated with mood disorders or antidepressants response [23]–[26]. However, no literature reported the association between those three *NTRK2* gene polymorphisms and the susceptibility to schizophrenia.

The most possible reason for inconsistent results may be that schizophrenia is characterized by heterogeneous clinical features; and different subtype of schizophrenia may lead to the genetic complexity of the disease [27]. Furthermore, the most important thing is that gene-gene interaction is critical to describe a phenotypic effect, when a specific individual genetic variant has a minor marginal effect in a complex psychiatric disease [28].

Thus, in present study, patients with paranoid schizophrenia (the most common subtype of schizophrenia) were recruited to improve the homogeneity of samples. The aim of the present study is to investigate the role of *BDNF* functional Val66Met polymorphism (rs6265), three polymorphisms in *NTRK2* gene (rs1387923, rs2769605 and rs1565445) and their interaction in pathophysiology of paranoid schizophrenia. To our knowledge this is the first study to investigate the genetic risk factors for the development of paranoid schizophrenia concerning gene-gene interaction.

## Materials and Methods

### Subjects

All procedures including standard informed consent were reviewed and approved by Institutional Review Boards of Shanghai Mental Health Center. This study was conducted in accordance with the Helsinki Declaration as revised 1989. All participants or their guardians (if the paptients had a compromised capacity) read the informed consent and were explained carefully about each item. Written informed consent was obtained from each participant or their guardians (if the paptients had a compromised capacity) before any study-related procedures were performed. All potential participants who declined to participate or otherwise did not participate were eligible for treatment and were not disadvantaged in any other way by not participating in the study.

All the subjects in this study undergo the Mini International Neuropsychiatric Interview (MINI) and were interviewed by two experienced psychiatrists. MINI is a brief structured interview for Axis I diagnosis of major psychiatric disorders in Diagnostic and Statistical Manual of Mental Disorders-Fourth Edition (DSM-IV) and International Classification of Diseases-Tenth Edition (ICD-10). Patients were recruited from Shanghai mental health center. Inclusion criteria were as follow: (1) Between the ages of 18 and 65; (2) Han Chinese in origin; (3) Meet the criteria of the Diagnostic and Statistical Manual of Mental Disorders, Fourth Edition (DSM-IV) for paranoid schizophrenia; (4) Blood samples were available. Patients who had a lifetime diagnosis of bipolar disorder, schizoaffective disorder, or other psychotic disorders were excluded.

Age and gender-matched healthy subjects were recruited excluding individuals with any major Axis I disorders and family history of mental disorder. Eventually, total 402 patients with a mean age of 34.9±5.4 years (230 males and 172 females) and matched 406 healthy controls with a mean age of 35.6±7.0 years (249 males and 157 females) were involved in this study. There were no significant difference of age and gender between cases and healthy subjects (t = 1.46, df = 806, p = 0.14; x^2^ = 1.42, df = 1, p = 0.23, respectively).

### DNA Extraction and SNP Genotyping

Peripheral blood samples were collected from the paticipants in 2 ml EDTA vacuum tube. Genomic DNA was extracted from peripheral blood according to standard laboratory procedures (Blood genomic DNA extraction kit, TIANGEN, Beijing) and stored in at −80°C until genotyping. The genotypes of the *BDNF* (rs6265) and *NTRK2* gene (rs1387923, rs2769605 and rs1565445) were identified as reported in our previous study [26]. For quality control, all genotypes were called blind to the case or control status in the genotyping process. Of the samples collected, 10% were repeated for the genotyping assay, and the results were more than 99% concordant.

### Statistical Analyses

Differences in age and gender between cases and control subjects were compared by using t-test and chi-square (χ2) test respectively. Hardy-Weinberg Equilibrium, genotype and allele frequencies of individual SNPs, pair-wise linkage disequilibrium of all pairs of SNPs and haplotype analysis were calculated by using the Haploview 4.1 software. The extent of linkage disequilibrium (LD) was measured by the standardized D’. Those haplotypes with a frequency under 3% were ignored. For multiple test correction, the Bonferroni correction was applied and the significance level α was set at 0.0125 (0.05/4). The power to detect significant association was estimated using the online software Quanto (Version 1.2.3, available at http://hydra.usc.edu/GxE/) [29]. The statistical power of our study was more than 90% under the assumption of a moderate effect size (Odds Ratio [OR] = 1.5), a log additive model and disease prevalence of 1%.

Gene-gene interactions were investigated by using multifactor-dimensionality reduction (MDR) method [30]. In brief, MDR combined higher and lower predisposing genotypes into two different groups (high or low-risk). Then the combination model is selected based on the lower misclassification error. Furthermore, 10-fold cross-validation was applied to assess the predictive ability of the each model by calculating the prediction error. Then, the best model with the maximization of cross-validation consistency was selected. P values of prediction accuracy were determined empirically by permuting the case and control labels 1,000 times. Hierarchical interaction graphs and interaction dendrogram of MDR were applied to present the SNPs interaction of the best model [31]. In addition, traditional statistical methods were performed to examine the results from MDR analyses. A *p* value of less than 0.05 was considered statistically significant.

## Results

### Hardy–Weinberg Equilibrium (HWE) and Linkage Disequilibrium (LD)

The distributions of genotypes in cases and control subjects were consistent with Hardy–Weinberg equilibrium (P>0.05) respectively. Analysis of pair wised LD was conducted for three SNPs in *NTRK2* gene. The D′ and r^2^ of three SNPs in *NTRK2* gene are shown in Table 1.

**Table 1:** Pairwise linkage disequilibrium results among SNPs in *NTRK2*.

	rs1387923	rs1565445	rs2769605
**rs1387923**		0.001	0.018
**rs1565445**	0.000		0.085
**rs2769605**	0.000	0.001	

D′ and r^2^ values are shown above and below the diagonal respectively.

### Association Analysis of Four Polymorphisms with Paranoid Schizophrenia

As shown in Table 2, no statistically significant differences were found in allele or genotype frequencies between cases and control subjects for four individual SNPs (rs1387923, rs1565445 and rs2769605 in *NTRK2* gene, rs6265 in *BDN*F gene). Haplotype-based analysis showed no significant association between the haplotypes from rs1387923, rs1565445 and rs2769605 in *NTRK2* and paranoid schizophrenia (detailed in Table 3).

**Table 2:** Allele and genotype distributions of SNPs and association analysis of each SNP between Case and Control samples.

SNPs	Sample	N	Genotype (%)			*χ2*	*P* a	Allele (%)		*χ^2^*	*P* a	OR (95% CI)
rs1387923			**T/T**	**T/C**	**C/C**			**T**	**C**			
	Case	402	237(59.0)	138(34.3)	27(6.7)	2.98	0.23	612(76.1)	192(23.9)	2.36	0.12	1.19 (0.95∼1.49)
	Control	406	2154(53.0)	161(39.7)	30 (7.4)			591(72.8)	221(27.2)			
rs1565445			**T/T**	**T/C**	**C/C**			**T**	**C**			
	Case	402	186(46.3)	169(42.0)	47(11.7)	1.30	0.52	541(67.3)	263(32.7)	1.25	0.26	0.89 (0.72∼1.09)
	Control	406	172(42.2)	181(44.6)	53(13.1)			525 (64.7)	287(35.3)			
rs2769605			**G/G**	**G/A**	**A/A**			**G**	**A**			
	Case	402	242(60.2)	132(32.8)	28(7.0)	2.03	0.36	616 (76.6)	188(23.4)	2.03	0.52	0.93 (0.73∼1.17)
	Control	406	246(60.6)	141(34.7)	19(4.7)			633(78.0)	179(22.0)			
rs6265			**G/G**	**G/A**	**A/A**			**G**	**A**			
	Case	402	119(29.6)	184(45.8)	99(24.6)	0.28	0.87	422(52.5)	382(47.5)	0.08	0.77	0.97 (0.80∼1.18)
	Control	406	120(29.6)	192(47.3)	94(23.2)			432(53.2)	380(46.8)			

a P-values are adjusted by Bonferroni method for the number of tests performed, the level of significance was set at 0.0125.

**Table 3:** Haplotype analysis of *NTRK2* gene (rs1387923–rs1565445–rs276905).

Gene	Haplotypers1387923–rs1565445–rs27690	Frequency (%)		*χ2*	*P* a	*OR* (95%CI)
		Case	Control			
*NTRK2*	T-C-G	154.14(19.2)	170.35(21.0)	0.89	0.35	0.89(0.70–1.14)
	T-C-A	41.74(5.2)	44.15(5.4)	0.06	0.81	0.95(0.61–1.47)
	T-T-G	311.38(38.7)	299.82(36.9)	0.49	0.48	1.08(0.88–1.32)
	T-T-A	104.74(13.0)	76.69(9.4)	5.10	0.02	1.43(1.05–1.96)
	C-C-G	54.26(6.7)	56.83(7.0)	0.05	0.83	0.96 (0.65–1.41)
	C-T-G	96.22(12.0)	106.01(13.1)	0.47	0.49	0.90(0.67–1.21)
	C-T-A	28.66(3.6)	42.48(5.2)	2.72	0.10	0.67(0.41–1.08)

Only haplotypes with frequency <0.03 are ignored in analysis.

a P-values are adjusted by Bonferroni method for the number of tests performed, the level of significance was set at 0.0125.

### MDR Analysis of Gene-gene Interaction

Cross-validation consistency and the prediction error obtained from MDR analysis for each number of loci were shown in Table 4. One three-locus model *BDNF* (rs6265)-*NTRK2* (rs1387923, rs2769605) had a maximum testing accuracy of 57.01% and a maximum cross-validation consistency (10/10) that was significant at *p*<0.0001 level, after determined empirically by permutation testing.

**Table 4:** The best model for predicting the occurrence of the paranoid schizophrenia.

Best model	Training accuracy (%)	Testing accuracy (%)	CVC	*x^2^*	*p* value	OR 95%CI
*NTRK2* (rs1387923)	53.01	50.87	9/10	2.95	0.09	1.28 (0.97–1.69)
*NTRK2* (rs1387923, rs1565445)	54.51	48.14	5/10	5.73	0.02	1.40 (1.06–1.85)
*NTRK2* (rs1387923, rs2769605), *BDNF* (rs6265)	57.01	53.09	10/10	15.61	<0.0001	1.75 (1.33–2.31)

CVC = Cross-validation consistency.

**Figure 1:**
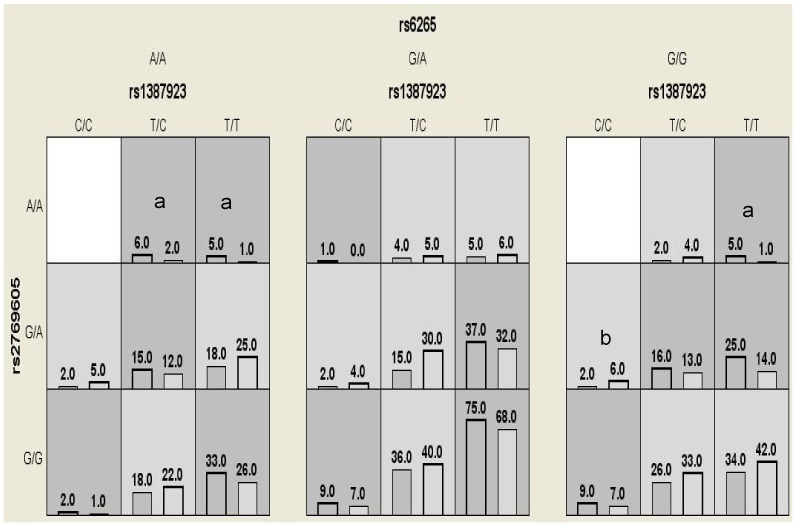
Distribution of high-risk and low-risk genotypes in the best three-locus model. Dark gray and light gray boxes presented the high- and low-risk factor combinations, respectively. Left bars within each box represented case while the right bars represented control. The heights of the bars are proportional to the sum of samples in each group. Note that the patterns of high-risk and low-risk cells differ across each of the different multilocus dimensions. This is evidence of epistasis, or gene-gene interaction. “a” and “b” represented high-risk and low-risk genotype combinations respectively which were also validated by traditional statistical analysis.

Figure 1 summarizes the three-locus genotype combinations associated with high risk or low risk for each multilocus-genotype combination. The varied patterns of high-risk and low-risk cells across each of the different multi-locus dimensions provide evidence of epitasis, or gene–gene interaction; that is, the influence that each genotype at a particular locus has on disease risk is dependent on the genotypes at each of the other two loci (30).

Traditional statistical methods were applied to this three-locus model to aid in interpretation, which identified three high-risk genotype combinations and one low-risk genotype combination from all possible genotype combinations. In this three-locus (rs1387923–rs2769605–rs6265) model, the OR for the low-risk genotype combination (CC)-(GA)-(GG) was 0.33 (95% CI: 0.18–0.67), the ORs for the three high-risk genotype combinations (TT)-(AA)-(AA), (TT)-(AA)-(GG), and (TC)- (AA)-(AA) were 5.1 (95% CI: 1.5–7.4), 5.1 (95% CI: 1.5–7.4) and 3.06 (95% CI: 1.3–5.8) respectively.

### Hierarchical Interaction Graphs

**Figure 2:**
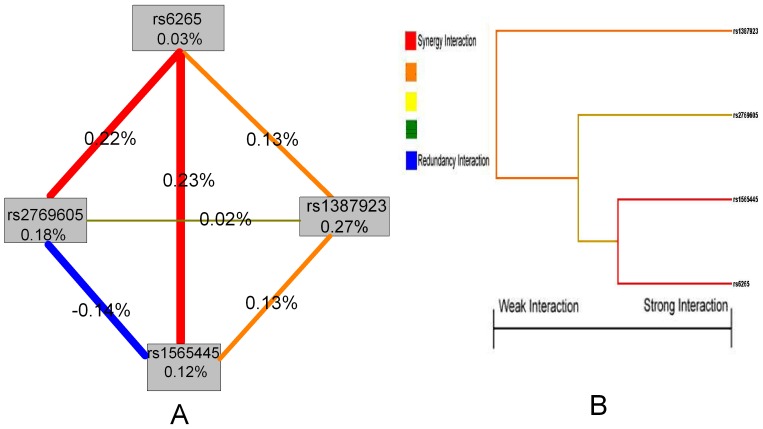
Hierarchical interaction graphs and interaction dendrogram. (A) Hierarchical interaction graphs showed that the percentage at the bottom of the each polymorphism represented entropy of it, and the percentage on each line represented the interaction percentage of entropy between two polymorphisms. The red line represented synergy redundancy interaction and the blue line represented redundancy interaction. (B) Interaction dendrogram showed that the red line represented synergy interaction and the orange line represented synergy interaction more weakly. From left to right the interaction was more intensive.

After identifying a high-risk combination of SNPs using MDR, we used the theory of information gain to interpret the relationship between these four SNPs and drew the hierarchical interaction graphs. As shown in Figure 2A, we found a positive interaction effect of rs6265 in *BDNF* gene and rs1565445 in *NTRK2* with interaction entropy of 0.23%; rs6265 in *BDNF* gene and rs2769605 in *NTRK2* with interaction entropy of 0.22%; rs6265 in *BDNF* gene and rs1387923 in *NTRK2* with interaction entropy of 0.13%; rs1565445 and rs1387923 in *NTRK2* with interaction entropy of 0.13%; a negative interaction effect of rs1565445 and rs2769605 in *NTRK2* with interaction entropy of −0.14%.

### Interaction Dendrogram

Interaction dendrogram demonstrated that rs6265 in *BDNF* gene and its receptor’s one polymorphism rs1565445 in *NTRK2* gene located on the same branch (Figure 2B). These two SNPs were estimated to have the strongest synergy interaction, as indicated visually by the red line. The rs1387923 in *NTRK2* gene was on a different branch, demonstrating a synergy interaction with its two polymorphisms in *NTRK2* gene (rs1565445 and rs2769605) and rs6265 in *BDNF* gene as indicated visually by the orange line.

## Discussion

Recently, BDNF and its high-affinity receptor, NTRK2, widely expressed in the adult brain, were both reported decreased in human postmortem of schizophrenia suggesting they were involved in pathophysiology of schizophrenia [4], [9], [14]. However, genetic associations between *BDNF* and schizophrenia had a contradicting result. Single nucleotide polymorphism rs6265 is the most common and critical functional genetic polymorphisms of the *BDNF* gene. Meanwhile, *BDNF* polymorphism rs6265 can influence activity-dependent secretion of BDNF [15]. Some previous studies have showed significant association between this polymorphism of *BDNF* gene and schizophrenia, but some showed no association between them. The most essential reason for the inconsistent results is that the heterogeneity of schizophrenia may contribute to the genetic complexity of the disease, like varied ethnic of samples and different subtype of schizophrenia [27].

Currently, Sun et al. reported the significant differences in the genotype distribution and allelic frequencies of the *BDNF* polymorphism rs6265 between schizophrenic patients in a Chinese Han population (n = 456) and controls (n = 483) [19]. However, most research and meta-analysis reported the negative association between *BDNF* polymorphism rs6265 and schizophrenia in a Chinese population. Xu et al. showed that no significant differences were found in allele or genotype or haplotype frequencies of *BDNF* polymorphism rs6265 between Chinese schizophrenic patient and controls; and their meta-analysis demonstrated that the this polymorphism did not contribute to the susceptibility to schizophrenia [22]. Wang et al. [32] and Sun et al. [33] sequentially reported no association between *BDNF* variants rs6265 and schizophrenia in a Chinese population. Recently, Zhang et al. also found no association between *BDNF* polymorphism rs6265 and the susceptibility to schizophrenia [34]. Yi et al. also demonstrated that there was no significant differences of genotype or allele distribution between early onset schizophrenic patients (onset before age 18) (n = 353) and controls (n = 394) in a Chinese Han population [35].

Paranoid schizophrenia is the most common type of schizophrenia, and little research studied the association between specific schizophrenia subtype and polymorphism rs6265 in *BDNF* gene. Recently, Suchanek et al. found no association between *BDNF* polymorphism rs6265 and the development of paranoid schizophrenia [36]. Until now, no literature reported the relationship between *BDNF* polymorphism rs6265 and paranoid schizophrenia in a Chinese Han population.

NTRK2, a high-affinity receptor of BDNF play a critical role in BDNF/NTRK2 pathway; and it has also been found decreased in postmortem of schizophrenic subjects [12]. Previous studies have demonstrated that three polymorphisms in *NTRK2* gene (rs2769605, rs1387923, and rs1565445) were associated with mood disorders [23]–[26]. However, up to date, no literature reported the correlation between *NTRK2* gene (rs2769605, rs1387923, and rs1565445) and schizophrenia.

In the present study, we recruited 402 patients with paranoid schizophrenia and 406 control subjects to examine the putative association between paranoid schizophrenia and polymorphisms in *BDNF* (rs6265) and *NTRK2* genes (rs1387923, rs2769605, and rs1565445) in a Chinese Han population. No statistically significant differences in allele and genotype frequencies were observed between cases and control participants for all these four polymorphisms separately. The haplotype analysis showed no association between haplotype of *NTRK2* genes (rs1387923, rs2769605, and rs1565445) and paranoid schizophrenia. However, we found the association between the interaction of *BDNF* and *NTRK2* with paranoid schizophrenia by using the MDR method followed by conventional statistical analysis. The best gene-gene interaction model identified was a three-locus model (*BDNF* rs6265, *NTRK2* rs1387923 and *NTRK2* rs2769605). In this model, one low-risk and three high-risk four-locus genotype combinations were identified. We speculate that from our findings, since schizophrenia is a complex psychiatric disease, the individual genetic variants may just display minor marginal effects on its pathogenesis, and are hard detected; or some of the components, such as BDNF and its receptor NTRK2, in development of schizophrenia may act synergistically in ways we don’t understand.

To the best of our knowledge, this is the first study to explore the correlation between *BDNF* (rs6265) and three NTRK2 gene polymorphisms and paranoid schizophrenia in a Chinese Han population; and the first time reporting the association between the interaction of *BDNF* and *NTRK2* polymorphisms and the development of paranoid schizophrenia. However, several concerns or limitations still need to be addressed. Firstly, it is noteworthy that the moderate sample size and lack of independent replication, the current results should be interpreted with caution and further studies of independent, multiple-center, large-scale samples should be conducted to validate our results. Secondly, other crucial genes in BDNF/NTRK2 signaling pathway were not examined in the present study. For example, Kawanishi et al. found that two genetic variants (−933T–>C and −413G–>A) in the promoter region of the cyclic adenosine monophosphate response element binding (*CREB*) gene were found only in schizophrenics, not in controls [37]. Thus, the possible role of other genes in the BDNF/NTRK2 signaling pathway and their interactions on susceptibility to schizophrenia should be further examined.

In conclusion, our findings implied that single polymorphism of rs6265 rs1387923, rs2769605, and rs1565445 in BDNF and NTRK2 did not demonstrate the association with the development of paranoid schizophrenia in a Han population, however, our finding suggested statistically significant role of interaction of *BDNF* and *NTRK2* genes polymorphisms (*BDNF*-rs6265, *NTRK2*-rs1387923 and *NTRK2*-rs2769605) in schizophrenia susceptibility.

## References

[1] Ross CA, Margolis RL, Reading SA, Pletnikov M, Coyle JT (2006) Neurobiology of schizophrenia. Neuron 52: 139–153. 17015232 10.1016/j.neuron.2006.09.015

[2] Miyanishi T, Sumiyoshi T, Higuchi Y, Seo T, Suzuki M (2013) LORETA current source density for duration mismatch negativity and neuropsychological assessment in early schizophrenia.PLoS One. 8: e61152. 10.1371/journal.pone.0061152 PMC3618448 23577204

[3] Buckley PF, Pillai A, Howell KR (2011) Brain-derived neurotrophic factor: findings in schizophrenia. Curr Opin Psychiatry 24: 122–127. 21248641 10.1097/YCO.0b013e3283436eb7

[4] Favalli G, Li J, Belmonte-de-Abreu P, Wong AH, Daskalakis ZJ (2012) The role of BDNF in the pathophysiology and treatment of schizophrenia. J Psychiatr Res 46: 1–11. 22030467 10.1016/j.jpsychires.2011.09.022

[5] Pandya CD, Kutiyanawalla A, Pillai A (2013) BDNF-TrkB signaling and neuroprotection in schizophrenia. Asian J Psychiatr 6: 22–28. 23380313 10.1016/j.ajp.2012.08.010 PMC3565158

[6] Lu W, Zhang C, Yi Z, Li Z, Wu Z, et al (2012) Association between BDNF Val66Met polymorphism and cognitive performance in antipsychotic-naïve patients with schizophrenia. J Mol Neurosci 47: 505–510. 22477643 10.1007/s12031-012-9750-4

[7] Zhang XY, Liang J, Chen da C, Xiu MH, Yang FD, et al (2012) Low BDNF is associated with cognitive impairment in chronic patients with schizophrenia. Psychopharmacology (Berl) 222: 277–284. 22274000 10.1007/s00213-012-2643-y

[8] Lee AH, Lange C, Ricken R, Hellweg R, Lang UE (2011) Reduced brain-derived neurotrophic factor serum concentrations in acute schizophrenic patients increase during antipsychotic treatment. J Clin Psychopharmacol 31: 334–336. 21508862 10.1097/JCP.0b013e31821895c1

[9] Issa G, Wilson C, Terry AV Jr, Pillai A (2010) An inverse relationship between cortisol and BDNF levels in schizophrenia: data from human postmortem and animal studies. Neurobiology of Disease 39: 327–333. 20451611 10.1016/j.nbd.2010.04.017

[10] Weickert CS, Hyde TM, Lipska BK, Herman MM, Weinberger DR, et al (2003) Reduced brain-derived neurotrophic factor in prefrontal cortex of patients with schizophrenia. Molecular Psychiatry 8 (6): 592–610. 10.1038/sj.mp.4001308 12851636

[11] Squinto SP, Stitt TN, Aldrich TH, Davis S, Bianco SM, et al (1991) trkB encodes a functional receptor for brain-derived neurotrophic factor and neurotrophin-3 but not nerve growth factor. Cell 65: 885–893. 1710174 10.1016/0092-8674(91)90395-F

[12] Weickert CS, Ligons DL, Romanczyk T, Ungaro G, Hyde TM, et al (2005) Reductions in neurotrophin receptor mRNAs in the prefrontal cortex of patients with schizophrenia. Mol Psychiatry 10: 637–650. 15940304 10.1038/sj.mp.4001678

[13] Hashimoto T, Bergen SE, Nguyen QL, Xu B, Monteggia LM, et al (2005) Relationship of Brain-Derived Neurotrophic Factor and Its Receptor TrkB to Altered Inhibitory Prefrontal Circuitry in Schizophrenia. The Journal of Neuroscience 25: 372–383. 15647480 10.1523/JNEUROSCI.4035-04.2005 PMC6725470

[14] Thompson Ray M, Weickert CS, Wyatt E, Webster MJ (2011) Decreased BDNF, trkB-TK+ and GAD67 mRNA expression in the hippocampus of individuals with schizophrenia and mood disorders. J Psychiatry Neurosci 36: 195–203. 21223646 10.1503/jpn.100048 PMC3080515

[15] Egan MF, Kojima M, Callicott JH, Goldberg TE, Kolachana BS, et al (2003) The BDNF val66met polymorphism affects activity-dependent secretion of BDNF and human memory and hippocampal function. Cell 112: 257–269. 12553913 10.1016/s0092-8674(03)00035-7

[16] Pezawas L, Verchinski BA, Mattay VS, Callicott JH, Kolachana BS, et al (2004) The brain-derived neurotrophic factor val66met polymorphism and variation in human cortical morphology. J Neurosci 24: 10099–10102. 15537879 10.1523/JNEUROSCI.2680-04.2004 PMC6730170

[17] Muglia P, Vicente AM, Verga M, King N, Macciardi F, et al (2003) Association between the BDNF gene and schizophrenia. Mol. Psychiatry 8: 146–147. 10.1038/sj.mp.4001221 12610646

[18] Neves-Pereira M, Cheung JK, Pasdar A, Zhang F, Breen G, et al (2005) BDNF gene is a risk factor for schizophrenia in a Scottish population. Mol. Psychiatry 10: 208–212. 10.1038/sj.mp.4001575 15630410

[19] Sun MM, Yang LM, Wang Y, Feng X, Cui KY, et al.. (2013) BDNF Val66Met polymorphism and anxiety/depression symptoms in schizophrenia in a Chinese Han population. Psychiatr Genet [Epub ahead of print]. 10.1097/YPG.0b013e328360c866 23532065

[20] Gratacòs M, González JR, Mercader JM, de Cid R, Urretavizcaya M, et al (2007) Brain-derived neurotrophic factor Val66Met and psychiatric disorders: meta-analysis of case-control studies confirm association to substance-related disorders, eating disorders, and schizophrenia. Biol. Psychiatry 61: 911–922. 10.1016/j.biopsych.2006.08.025 17217930

[21] Kanazawa T, Glatt SJ, Kia-Keating B, Yoneda H, Tsuang MT (2007) Meta-analysis reveals no association of the Val66Met polymorphism of brain-derived neurotrophic factor with either schizophrenia or bipolar disorder. Psychiatry Genet 17: 165–170. 10.1097/YPG.0b013e32801da2e2 17417060

[22] Xu MQ, St Clair D, Ott J, Feng GY, He L (2007) Brain-derived neurotrophic factor gene C-270T and Val66Met functional polymorphisms and risk of schizophrenia: a moderate-scale population-based study and meta-analysis. Schizophr Res 91: 6–13. 17289348 10.1016/j.schres.2006.12.008

[23] Bremer T, Diamond C, McKinney R, Shehktman T, Barrett TB, et al (2007) The pharmacogenetics of lithium response depends upon clinical co-morbidity. Mol Diagn Ther 11: 161–170. 17570738 10.1007/BF03256238

[24] Smith EN, Bloss CS, Badner JA, Barrett T, Belmonte PL, et al (2009) Genome-wide association study of bipolar disorder in European American and African American individuals. Mol Psychiatry 14: 755–763. 19488044 10.1038/mp.2009.43 PMC3035981

[25] Wang Z, Li Z, Gao K, Fan J, Wang L, et al (2012) Association of BDNF Gene Polymorphism with Bipolar Disorder in Han Chinese Population. Genes Brain Behav 11: 524–528. 22548711 10.1111/j.1601-183X.2012.00797.x

[26] Li Z, Zhang Y, Wang Z, Chen J, Fan J, et al (2013) The role of BDNF, NTRK2 gene and their interaction in development of treatment-resistant depression: data from multicenter, prospective, longitudinal clinic practice. J Psychiatr Res 47: 8–14. 23137999 10.1016/j.jpsychires.2012.10.003 PMC3584686

[27] Zhang C, Li Z, Shao Y, Xie B, Du Y, et al (2011) Association study of tryptophan hydroxylase-2 gene in schizophrenia and its clinical features in Chinese Han population. J Mol Neurosci 43: 406–411. 20938755 10.1007/s12031-010-9458-2

[28] Burmeister M, McInnis MG, Zollner S (2008) Psychiatric genetics: progress amid controversy. Nat Rev Genet 9: 527–540. 18560438 10.1038/nrg2381

[29] Gauderman WJ, Morrison JM (2006) QUANTO 1.1: A computer program for power and sample size calculations for genetic-epidemiology studies. http://hydra.usc.edu/gxe. Accessed 2009 May.

[30] Ritchie MD, Hahn LW, Roodi N, Bailey LR, Dupont WD, et al (2001) Multifactor-dimensionality reduction reveals high-order interactions among estrogen-metabolism genes in sporadic breast cancer. Am J Hum Genet 69: 138–147. 11404819 10.1086/321276 PMC1226028

[31] Dervieux T, Wessels JA, Kremer JM, Padyukov L, Seddighzadeh M, et al (2012) Patterns of interaction between genetic and nongenetic attributes and methotrexate efficacy in rheumatoid arthritis. Pharmacogenet Genomics 22: 1–9. 22044941 10.1097/FPC.0b013e32834d3e0b

[32] Wang Y, Wang JD, Wu HR, Zhang BS, Fang H, et al (2010) The Val66Met polymorphism of the brain-derived neurotrophic factor gene is not associated with risk for schizophrenia and tardive dyskinesia in Han Chinese population. Schizophr Res 120: 240–242. 20395113 10.1016/j.schres.2010.03.020

[33] Sun RF, Zhu YS, Kuang WJ, Liu Y, Li SB (2011) The G-712A polymorphism of brain-derived neurotrophic factor is associated with major depression but not schizophrenia. Neurosci Lett 489: 34–37. 21129438 10.1016/j.neulet.2010.11.061

[34] Zhang XY, Chen da C, Xiu MH, Haile CN, Luo X, et al (2012) Cognitive and serum BDNF correlates of BDNF Val66Met gene polymorphism in patients with schizophrenia and normal controls. Hum Genet 131: 1187–1195. 22362486 10.1007/s00439-012-1150-x PMC3671849

[35] Yi Z, Zhang C, Wu Z, Hong W, Li Z, et al (2011) Lack of effect of brain derived neurotrophic factor (BDNF) Val66Met polymorphism on early onset schizophrenia in Chinese Han population. Brain Res 1417: 146–150. 21917241 10.1016/j.brainres.2011.08.037

[36] Suchanek R, Owczarek A, Paul-Samojedny M, Kowalczyk M, Kucia K, et al (2013) BDNF val66met polymorphism is associated with age at onset and intensity of symptoms of paranoid schizophrenia in a Polish population. J Neuropsychiatry Clin Neurosci 25: 88–94. 23487199 10.1176/appi.neuropsych.11100234

[37] Kawanishi Y, Harada S, Tachikawa H, Okubo T, Shiraishi H (1999) Novel variants in the promoter region of the CREB gene in schizophrenic patients. J Hum Genet 44: 428–430. 10570922 10.1007/s100380050196

